# Metabolomic profile of cerebral tissue after acoustically-mediated blood-brain barrier opening in a healthy rat model: a focus on the contralateral side

**DOI:** 10.3389/fnmol.2024.1383963

**Published:** 2024-11-20

**Authors:** Antoine Presset, Sylvie Bodard, Antoine Lefèvre, Anaïs Millet, Edward Oujagir, Camille Dupuy, Tarik Iazourène, Ayache Bouakaz, Patrick Emond, Jean-Michel Escoffre, Lydie Nadal-Desbarats

**Affiliations:** ^1^UMR 1253, iBrain, Inserm, Université de Tours, Tours, France; ^2^Département Analyses Chimique et Métabolomique, PST Analyses des Systèmes Biologiques, Université de Tours, Tours, France; ^3^CHRU Tours, Serv Med Nucl in Vitro, Tours, France

**Keywords:** blood–brain barrier opening, metabolomics, brain’s contralateral side, amino acid metabolisms, compensatory mechanism, ultrasound, microbubbles

## Abstract

Microbubble (MB)-assisted ultrasound (US) is an innovative modality for the non-invasive, targeted, and efficient delivery of therapeutic molecules into the brain. Previously, we reported the first metabolomic signature of blood–brain barrier opening (BBBO) induced by MB-assisted US. In the present study, the neurometabolic consequences of acoustically-mediated BBBO on cerebral tissue were investigated using multimodal metabolomics approaches. Sinusoid US waves (1 MHz, peak negative pressure 0.6 MPa, burst length 10 ms, total treatment time 30 s, MB bolus dose 0.7 × 10^5^ MBs/g) were applied on the rats’ right striatum (ipsilateral side). Brain was collected and both striata were then dissected 3 h, 2 days, and 1 week after BBBO. After tissue preparation, the samples were analyzed using nuclear magnetic resonance spectrometry (NMRS) and high-performance liquid chromatography coupled to mass spectrometry (HPLC-MS). Our findings showed a slight disruption of metabolic pathways in contralateral striata of animals. Analyses of metabolic pathways indicated changes in amino acid metabolisms. In addition, tryptophan derivate dosages revealed the perturbation of a central metabolite of the kynurenine pathway (i.e., 3-hydroxy-kynurenine). In conclusion, the acoustically-mediated BBBO of the ipsilateral cerebral hemisphere induced significant change in metabolism of contralateral one.

## 1 Introduction

**Figure 1:**
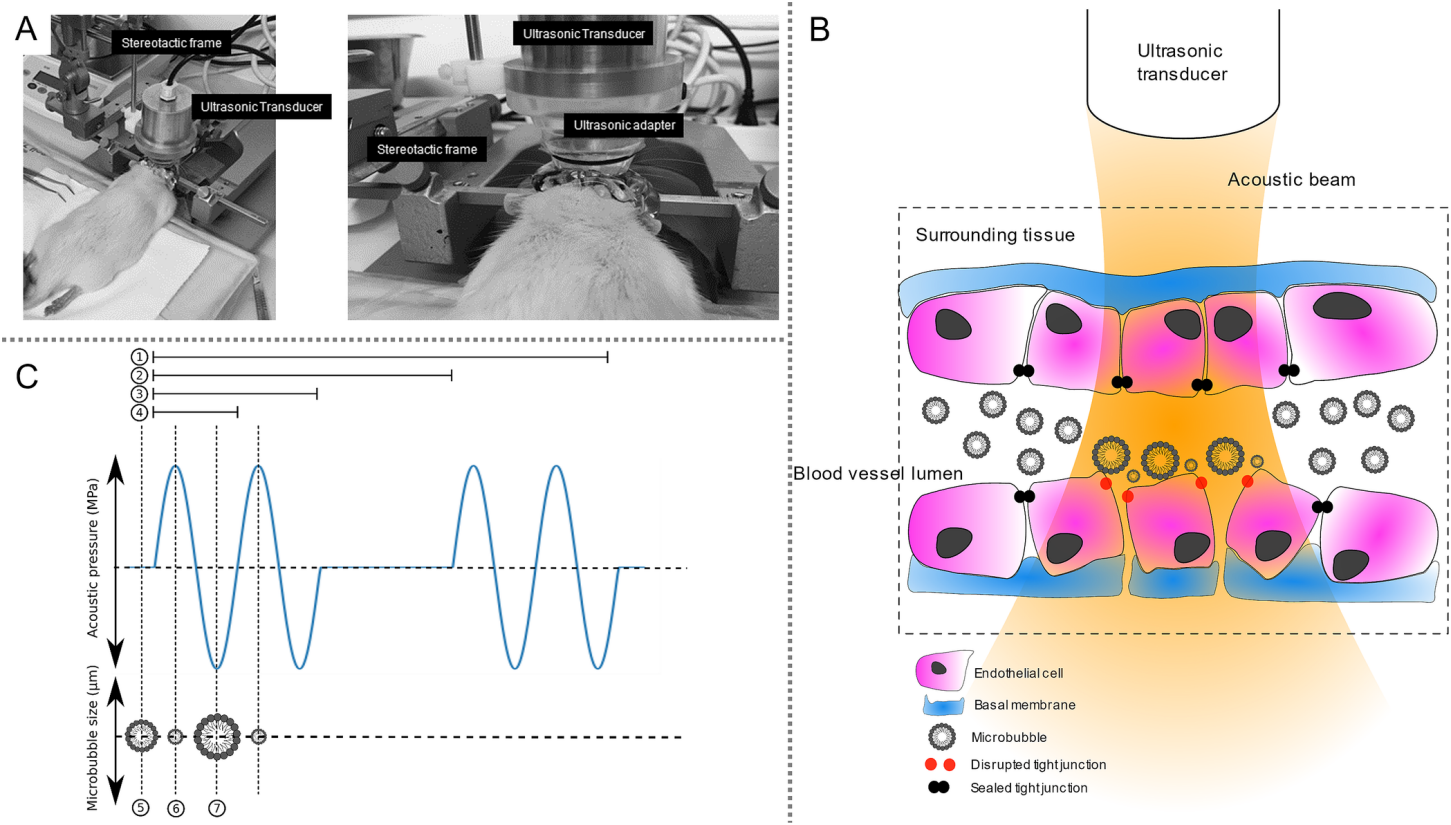
Experimental setup for blood–brain barrier opening by microbubble-assisted ultrasound and its main biological effect. **(A)** Experimental setup. The rat was positioned in stereotaxic frame and a preparative surgery was done. Then, an ultrasonic transducer was placed over the striatum according to stereotaxic coordinates. **(B)** Physical and biological processes leading to the opening of the blood–brain barrier. In an acoustic field, microbubbles injected into the blood circulation are pushed along the apical vascular wall and oscillate at their nominal frequency. These oscillations cause transient disruptions of tight junctions, thereby increasing the permeability of the BBB. **(C)** Example of a waveform of pulsed ultrasonic emission. 1 represents the exposure duration (i.e., 30 s); 2 represents the pulse repetition duration (i.e., 1 s); 3 represents the burst length (i.e., 10 ms); 4 represents the period of the sinusoidal wave (1 μs, corresponding to the frequency of 1 MHz). When no ultrasound is applied, microbubbles have their nominal diameter (5). When a positive peak of the ultrasound wave occurs, it corresponds to a compression phase leading to the shrinking of microbubbles (6). Then, a positive peak of the ultrasound wave occurs, leading to rarefaction causing expansion of microbubbles (7).

Microbubble-assisted ultrasound (MB-assisted US) is a promising modality for the efficient, safe, and targeted intracerebral (i.c.) delivery of therapeutics. This US technology relies on the intravenous administration of MBs and the transcranial application of focused US, which locally activates these MBs (Figure 1). This activation increases the native permeability of cerebrovascular endothelium (also termed blood–brain barrier), thus improving the extravasation and the bioavailability of therapeutics in the targeted brain tissue (Hughes et al., 2023; McMahon et al., 2021; Presset et al., 2020). MB-assisted US allows the i.c. delivery of wide range of therapeutics including chemotherapeutics, immunotherapeutics, nucleic acids, and cells (e.g., stem or immune cells). The blood–brain barrier opening (BBBO) can be temporally and spatially controlled, thus resulting in the non-invasive targeting of either superficial or deep brain regions. MB-assisted US is presently attracting great medical interest for the treatment of wide range of brain disorders such as neurodegenerative diseases (e.g., Alzheimer disease, Parkinson disease, amyotrophic lateral sclerosis, etc.), neuropsychiatric disorders (e.g., depression, dementia etc.), and brain tumors (e.g., primary tumors and metastases) (Abrahao et al., 2019; Lipsman et al., 2018; Meng et al., 2022; Shin et al., 2018; Wang et al., 2020).

The innocuity of MB-assisted US has been well-investigated through a complementary and multidisciplinary studies including cellular and molecular biology, transcriptomics, histology, *in vivo* imaging (e.g., magnetic resonance imaging and positron emission tomography) and animal behavior (McMahon et al., 2019; Meng et al., 2019). All these studies together convey that MB-assisted US leads to low or no neurophysiological damage onto the targeted tissue when this modality is rationally used. Indeed, such damages are only observed when inappropriate US and MB-related parameters were applied for an efficient and safe drug delivery (McMahon et al., 2020a; McMahon et al., 2020b; McMahon et al., 2017, 2018, 2019; McMahon and Hynynen, 2017).

In our previous work, we characterized the peripheral and central metabolic responses of acoustically-mediated BBBO for the first time (Presset et al., 2022). In this study, the dysregulations of metabolic pathways in blood serum, in cerebrospinal fluid, in urine (peripheral biological matrices), and in ipsilateral striata (central biological matrix) were explored using multimodal and multi-matrix metabolomics approaches. The most important change in metabolic response to the BBBO was detected in the vascular circulation associated with a transient vascular inflammation. In addition, significant changes in arginine and arginine-related metabolisms were observed in all matrices after BBBO, highlighting an activation of vasomotor processes and bioenergetic supply. The serotonergic neurotransmission pathways were also transiently disrupted in the ipsilateral striatum after BBBO. These metabolic profiles in central and peripheral biological matrices were not related to physiological signs of suffering. In this previous study, we did not explore the influence of acoustically-mediated BBBO on the metabolism of contralateral striata. The striatum is a paired functional structure in the brain involved in motor and cognitive functions, including in the pathophysiology of Parkinson’s disease. Consequently, the objective of the present study was to investigate the metabolic consequences of BBBO in ipsilateral striatum on the contralateral striatum using the same analytical workflow by means of nuclear magnetic resonance spectrometry (NMRS) and high-performance liquid chromatography coupled to mass spectrometry (HPLC-MS).

## 2 Materials and methods

### 2.1 Acoustically-mediated blood–brain barrier opening

All male rats Sprague Dawley (Janvier Labs, Le Genest-Saint-Isle, France) were 7 weeks old (about 250 g) before experiments. They were housed in groups of 4 under humidity and temperature-controlled conditions and a 12:12 light–dark cycle (light on at 7:00 AM) with *ad libitum* access to food and water. Animals were acclimated to their housing for 1 week before the *in vivo* procedures.

The BBB of the right striatum (called ipsilateral) was acoustically permeabilized using the US protocol and setup reported in Presset et al. (2022). In brief, the anesthetized animals were positioned into a stereotaxic frame and a heating plate. The coordinates of the bregma are recorded, and then the relative coordinates of the striatum are derived. The US probe was then placed at the relative stereotactic coordinates of the striatum. After an intravenous bolus injection of MBs (Vevo MicroMarker MM-1, 100 μL at 2.5 × 10^8^ MBs/mL; Fujifilm-Visualsonics Inc., Amsterdam, the Netherlands), the striatum was exposed to 1 MHz sinusoid US waves with a pulse repetition frequency of 1 Hz, 10,000 cycles per pulse (10 ms burst length) at a peak negative pressure of 0.6 MPa for 30 s. This commonly used US protocol induces a transient BBBO without inducing hemorrhage through mechanical acoustic cavitation effects of MBs (Kovacs et al., 2017; McMahon and Hynynen, 2017; McMahon et al., 2017). Finally, the animals were sacrificed 3 h (3-h group), 2 days (2-day group), and 1 week (1-week group) after the BBBO for metabolomics analysis. Animals from control groups were treated as described above without US exposure.

### 2.2 Metabolomics analysis

After an intracardiac perfusion of saline solution, the contralateral (left) and ipsilateral (right) striata were dissected and then lyophilized and homogenized. Dry residues were weighted (about 3 mg). Finally, metabolites were extracted according to the protocol defined by Diémé et al. (2017). Samples were analyzed by using (i) ^1^H-NMR with a Bruker DRX-600 Avance III HD spectrometer (Bruker, Billerica, MA, United States) equipped with a TCI cryoprobe as previously described (Presset et al., 2022) and by using (ii) HPLC-MS with UHPLC Ultimate WPS-3000 system (Dionex, Sunnyvale, CA, United States) coupled to a Q-Exactive mass spectrometer (Thermo Fisher Scientific, Bremen, Germany). Finally, tryptophan metabolism intermediates were quantitatively measured using the HPLC-MS protocol described in Lefevre et al. (2019).

As previously described by Presset et al. (2022), all metabolomic data were treated in well-established bio-informatic workflow including peak identification, shift displacement interpretation, mass and retention time couples’ identification, integration/quantification, and metabolite annotations (Presset et al., 2022). Then multivariate and univariate data analyses were performed using ropls R package 1.22 (Thevenot et al., 2015) in order to determine (i) metabolic profiles and/or (ii) differentially expressed metabolites between our different groups to achieve metabolic pathways analysis (For details, Presset et al., 2022). PCA is commonly used for dimensionality reduction by projecting each data point onto only the first few principal components to obtain lower-dimensional data while preserving as much of the data’s variation as possible. It allows to summarize data by reducing the number of variables and denoising the data. In addition, this statistical approach has several goals: (i) exploration of large dataset characterized by several quantitative variables; (ii) identification of cluster by grouping samples that share a nearly metabolic profiles, represented by score plot; and (iii) identification of aberrant samples called outliers.

## 3 Results

In our experimental conditions, the Evans Blue assay revealed that MB-assisted US induced the BBBO in the ipsilateral striatum only and not in the contralateral one (Presset et al., 2022). The contralateral striata were then collected at different time points after acoustically- mediated BBBO for metabolomic analyses (Table 1).

**Table 1:** Sample size for metabolic exploration of contralateral striata after blood–brain barrier opening.

Groups	Control	3-h	2-day	1-week
# of striata	8	8	8	8
Outliers	0	1	0	1
Data included	8	7	8	7

**Figure 2:**
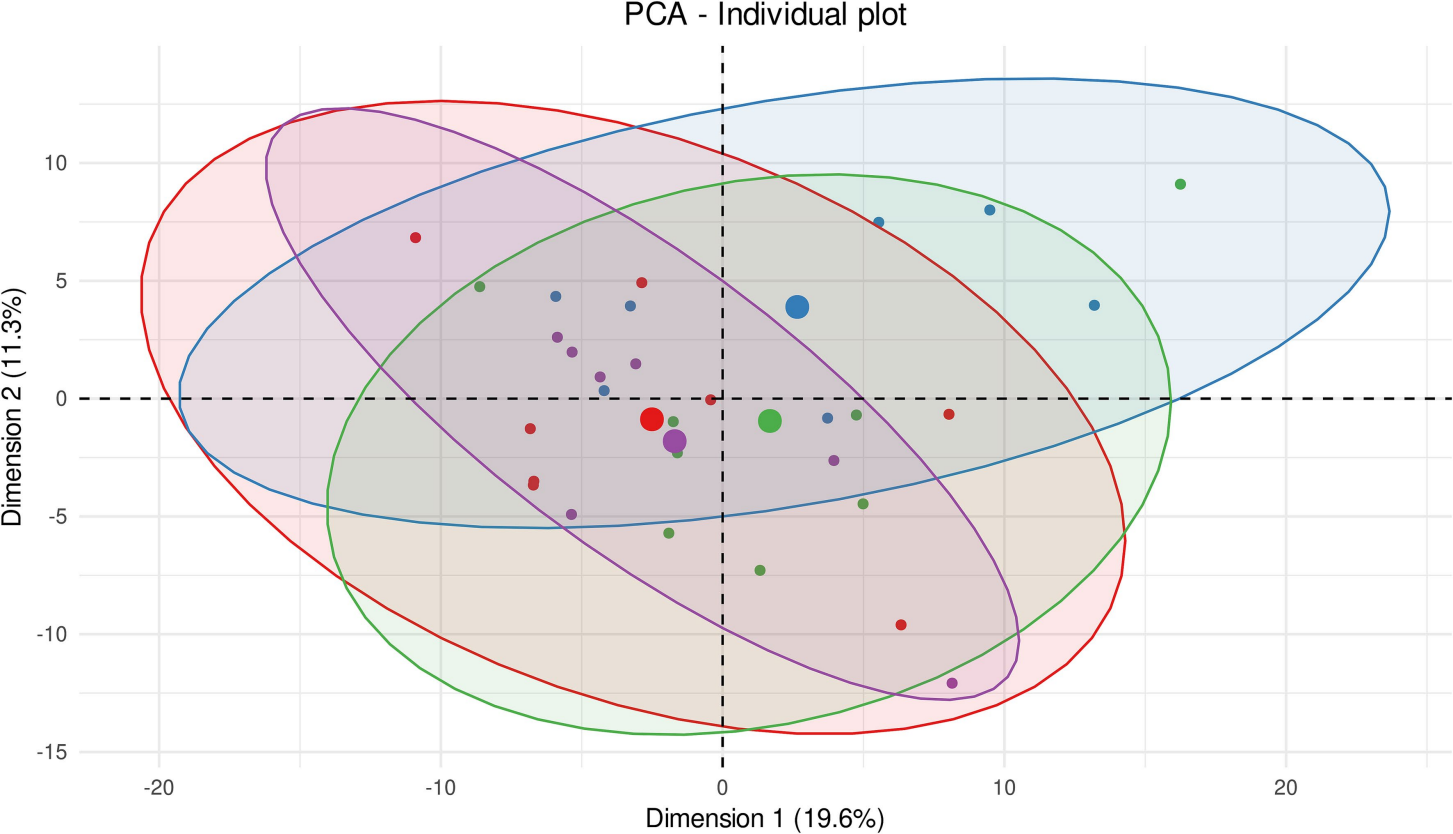
Unsupervised multivariate analyses on contralateral striatum metabolome between control and insonified groups. Score plot of principal component analysis (PCA) constructed with metabolites within the contralateral striatum metabolome after acoustically-mediated BBBO. Larger dots are inertial centers of ellipses (center of a sample group) plotted based on smaller dots representing each sample individually. Red dots are control samples; Blue dots are 3-h post-BBBO samples; Green dots are 2-day post-BBBO samples; Purple dots are 1-week post-BBBO samples. Two samples having the same metabolic profile will be clustered in the same dimensional space.

The principal component analyses (PCA) showed the homogeneity of metabolomic profiles from contralateral striata between control, 2-day and 1-week experimental groups (Figure 2). The inertial centers of the ellipses (center of a sample group) plotted based on smaller dots, representing each sample individually, were clustered all together in the center of the score plot. This result thus reveals that these three groups share an identical metabolic profile. It should be noted that the metabolic profile of the contralateral striata of individuals from 3-h post-BBBO group induced a shift of the inertial center compared to the other groups. This shift may indicate a potential metabolic dysregulation happening 3 h after BBBO in the striatum. Nevertheless, the ellipses formed by all individuals from each group showed significant overlap, thus highlighting a high similarity in metabolomic profile of the contralateral striatum between the experimental groups.

Moreover, supervised partial least square discriminant analyses (PLS-DA) were performed to establish predictive models between two-time groups by constraining the sample membership to specific groups. The results of these analyses failed (failure of permutation tests, *p* value <0.05) to establish predictive models (Table 2).

**Table 2:** Summary of supervised multivariate partial least square discriminant analysis (PLS-DA) models computed from metabolites found in contralateral striatum metabolome differentiating two groups.

Models	R2X	R2Y	Q2	pR2Y	pQ2	VIP > 1
Control vs. 3-h	0.364	0.982	0.480	0.10	0.10	91
3-h vs. 2-day	0.415	0.985	0.782	0.15	**0.05**	106
2-day vs. 1-week	0.416	0.974	0.371	0.30	0.40	106
Control vs. 1-week	0.382	0.963	0.260	0.65	0.60	107
Control vs. 2-day	0.384	0.981	0.479	0.30	0.25	102
3-h vs. 1-week	0.372	0.981	0.160	0.15	0.25	69

Model is considered as significant if permutation metrics (pR2Y and pQ2, corresponding to the *p* value of permutation test) are inferior to 0.05 (in bold), as well as predictive metric (Q2) is higher than 0.5. Variable importance in projection (VIP) is exploitable for metabolic pathway analyses if and only if the predictive model is significant. Only VIP superior to 1 has been counted (VIP > 1 column).

**Figure 3:**
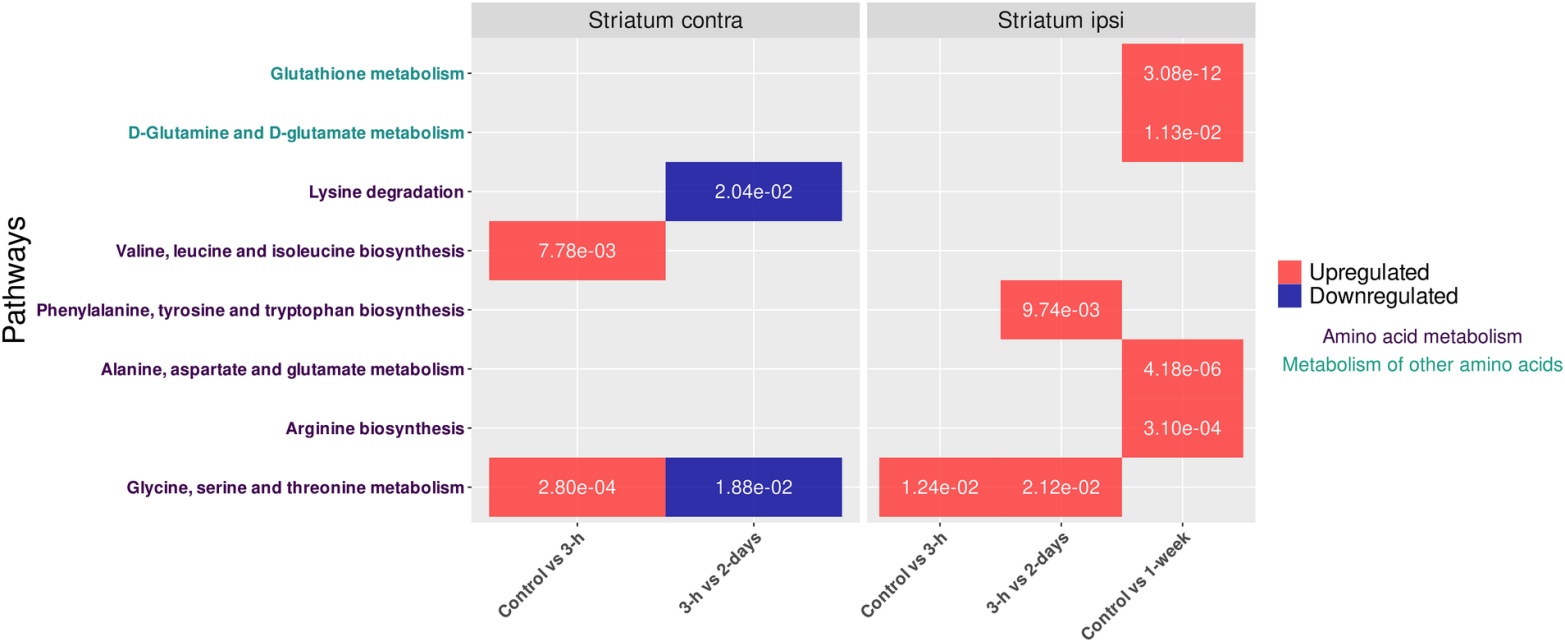
Impacted pathways in striata metabolomes of both cerebral hemispheres after acoustically-mediated BBBO. The adjusted *p* value is written into tiles. Dysregulation is represented in red and blue, respectively for upregulation and downregulation of the considered pathway. Pathway names are coded with colors depending on their metabolic classes.

Then, univariate analyses were achieved between all our experimental groups to determine differential production of metabolites. These analyses highlighted the significant variation in the intensities of 24 metabolites (over 236 detected and identified metabolites in contralateral striatum) despite the homogeneity of the metabolic profiles determined by both multivariate analyses (i.e., PCA and PLS-DA). Thus, metabolic pathway analysis with these significantly dysregulated metabolites revealed the disruption of (i) glycine, serine, and threonine metabolism, (ii) valine, leucine, and isoleucine biosynthesis and (iii) lysine degradation (Figure 3). Indeed, upregulation of glycine, serine, and threonine metabolism but also valine, leucine, and isoleucine biosynthesis were observed between control and 3-h post-BBBO groups. The glycine, serine and threonine metabolism returned to normal level 2 days after BBBO. In the same time interval (i.e., up to 2 days after BBBO), this metabolism is upregulated in ipsilateral striatum 3-h after BBBO (Presset et al., 2022). Conversely, this metabolism is downregulated in contralateral striatum between 3-h and 2-day groups after BBBO while it is upregulated in ipsilateral striatum. Finally, the lysine degradation pathway was downregulated in contralateral striatum between the 3-h and 2-day groups post-BBBO groups.

Finally, the dosage of tryptophan derivates showed a significant change in the concentration of only one tryptophan derivate. Indeed, 3-hydroxy-kynurenine concentration significantly decreased between 3 h and 2 days after BBBO (2.426 ± 1.239 vs. 1.356 ± 0.517 nmol/g) in the contralateral striatum.

## 4 Discussion

The present investigation aimed to determine the metabolic consequence of acoustically-mediated BBBO of ipsilateral striatum on the contralateral striatum using a well-established metabolomic workflow (Presset et al., 2022). Firstly, our metabolomic data revealed a slight disruption of metabolic pathways in the contralateral striatum within the same time interval, suggesting the establishment of a compensatory mechanism between both brain hemispheres. These dysregulated metabolic pathways were (i) the glycine, serine, and threonine metabolism, (ii) the valine, leucine, and isoleucine biosynthesis, and (iii) the lysine degradation. Moreover, the upregulation of glycine, serine, and threonine metabolism in both contralateral (present study) and ipsilateral (Presset et al., 2022) striata suggests a global disruption within the brain tissue after acoustically-assisted BBBO in our experimental conditions. Glycine is an amino acid neurotransmitter, which plays a major role in inhibitory synapses through the glycine receptor, as well as in excitatory synapses through the N-methyl-D-aspartate receptor (Marques et al., 2020). Additionally, serine is an amino acid involved in the synthesis of phospholipids and sphingolipids, whose metabolism is also related to that of glycine. Indeed, glycine is converted to serine, then to ethanolamine, and finally to phosphatidylethanolamine. This latter then undergoes three methylations to form phosphatidylcholine, and ultimately choline. The choline is next acetylated to produce acetylcholine, which participates in cholinergic neurotransmission. This neurotransmission is present in the striatum and represents 2% of the neurons (Kljakic et al., 2017; Lim et al., 2014). Therefore, the interhemispheric disruption of glycine, serine, and threonine metabolism could constitute a compensatory mechanism as well as a disruption of cholinergic neurotransmission, starting from 2 days after acoustically-mediated BBBO (i.e., upregulation in the ipsilateral striatum vs. downregulation in the contralateral striatum). These results could be confirmed by further PET/CT studies using radiotracers dedicated to the investigation of the cholinergic neurotransmission (Tiepolt et al., 2022).

Moreover, valine, leucine and isoleucine biosynthesis and lysine degradation metabolisms were also, respectively, dysregulated in the contralateral striatum after the acoustically-mediated BBBO of ipsilateral one. Valine, leucine and isoleucine are essential amino acids, which are required for protein synthesis in the brain, as in other tissues. As branched-chain amino acids (BCAA), they play a crucial role in cerebral metabolism (Sperringer et al., 2017). Indeed, BCAA can be converted into neurotransmitters, such as glutamate and then could produce the inhibitory neurotransmitter *γ*-aminobutyric acid (GABA). In addition, BCAA are involved in energy production in the brain. They can be metabolized to form intermediate compounds in the urea cycle and the Krebs cycle, thus contributing to the energy metabolism within the brain (Sperringer et al., 2017). BCAA contribute also to nitrogen balance in the brain by providing nitrogen for protein synthesis through glutamate and glutamine biosynthesis, thus regulating ammonia levels (Dodd et al., 1992; Holeček, 2018; Yudkoff et al., 2007). The upregulation of the biosynthesis metabolism of valine, leucine, and isoleucine 3 h after the BBBO may be a result of increased demand for energy metabolism or oxidative stress on the contralateral side to the BBB disruption. In addition, the lysine degradation is involved in the production of intermediates that can enter the tricarboxylic acid cycle (Krebs cycle), thus being implicated in the energy metabolism of cells. Furthermore, lysine degradation leads to the production of carnitine, which is involved in the transport of acetyl-CoA from the cytosol to the mitochondrial matrix for the catabolism of fatty acids (Matthews, 2020). The downregulation of the lysine degradation occurring between 3 h and 2 days after the acoustically-mediated BBBO could reveal a reduction of energetic demand or a diminution of catabolism of fatty acids.

The comparative study of metabolomic profiles from contralateral striata between insonified and control animals (i.e., without US exposure) revealed some disrupted metabolic pathways. Indeed, the analysis of the deregulated metabolic pathways highlighted the disruption of the brain metabolism from 3-h group in the contralateral striata of insonified animals compared to the control ones. These data emphasize (i) the importance of having a relevant “sham” control and (ii) that comparing the results obtained between both striata can lead to a misinterpretation of the neurometabolomic consequences of acoustically-mediated BBBO. It is important to keep in mind that metabolic perturbations in one brain hemisphere could be propagated to the other one through central or peripheral pathways. Therefore, the interpretation of internal comparisons, i.e., each animal is its own control, should be taken with the utmost caution. Moreover, this preliminary study did not establish if the metabolic changes observed in the contralateral striatum are the direct or indirect consequences of the acoustically-mediated BBBO in the ipsilateral striatum through neural circuitry or other physiological pathways. As a consequence, future investigations are required to identify the underlying mechanisms involved these metabolic changes using brain imaging, electrophysiological tools as electroencephalography, but also other omics approaches such as transcriptomics and proteomics. In addition, these metabolic changes might affect neurological and psychiatric outcomes. In the near future, it should be relevant to determine whether such metabolic changes take place in mouse models of neurological and neuropsychiatric disorders and whether these changes could influence the pathophysiological mechanisms and the symptoms of these disorders using brain imaging and behavior tests. Then, these metabolic changes could also have a significant impact on pharmacological treatments by disrupting the cerebral metabolism of drugs and/or the signaling pathways on which the drugs act. This hypothesis could be explored by analyzing the drug metabolism in brain tissues after its acoustically-mediated delivery using mass spectrometry and by investigating the signaling pathways involved in the therapeutic efficacy of the drugs using omics approaches.

## 5 Conclusion

In conclusion, the BBBO in the ipsilateral striatum resulted in minimal, transient but significant metabolomic changes in the contralateral striatum in our experimental conditions. The simultaneous dysregulation of an amino acid metabolism pathway (i.e., glycine, serine, and threonine metabolism) on both the ipsilateral and contralateral striata may suggest a compensatory phenomenon. These results highlight the importance of comparing two independent groups (i.e., sham vs. treated) when investigating the metabolic consequences of pharmacological or physical treatment (e.g., MB-assisted US) on the brain tissue. Moreover, further investigations are still required to evaluate the influence of acoustically-mediated BBBO on the metabolism of different brain regions of ipsilateral but also contralateral hemispheres.

## Data Availability

The datasets presented in this study can be found in online repositories. The names of the repository/repositories and accession number(s) can be found below: the datasets of this study are available in the online repository, Zenodo.org, with the DOI: 10.5281/zenodo.8420770 (Presset et al., 2023).
